# Causal Association Between Heart Failure and Alzheimer’s Disease: A Two-Sample Bidirectional Mendelian Randomization Study

**DOI:** 10.3389/fgene.2021.772343

**Published:** 2022-01-11

**Authors:** Chenglin Duan, Jingjing Shi, Guozhen Yuan, Xintian Shou, Ting Chen, Xueping Zhu, Yihan Yang, Yuanhui Hu

**Affiliations:** ^1^ Guang’anmen Hospital, China Academy of Chinese Medical Sciences, Beijing, China; ^2^ Beijing University of Chinese Medicine, Beijing, China

**Keywords:** Mendelian randomization, heart failure, Alzheimer’s disease, genome-wide association study, single-nucleotide polymorphisms, causal effects

## Abstract

**Background:** Traditional observational studies have demonstrated an association between heart failure and Alzheimer’s disease. The strengths of observational studies lie in their speed of implementation, cost, and applicability to rare diseases. However, observational studies have several limitations, such as uncontrollable confounders. Therefore, we employed Mendelian randomization of genetic variants to evaluate the causal relationships existing between AD and HF, which can avoid these limitations.

**Materials and Methods:** A two-sample bidirectional MR analysis was employed. All datasets were results from the UK’s Medical Research Council Integrative Epidemiology Unit genome-wide association study database, and we conducted a series of control steps to select the most suitable single-nucleotide polymorphisms for MR analysis, for which five primary methods are offered. We reversed the functions of exposure and outcomes to explore the causal direction of HF and AD. Sensitivity analysis was used to conduct several tests to avoid heterogeneity and pleiotropic bias in the MR results.

**Results:** Our MR studies did not support a meaningful causal relationship between AD on HF (MR-Egger, *p* = 0.634 > 0.05; weighted median (WM), *p* = 0.337 > 0.05; inverse variance weighted (IVW), *p* = 0.471 > 0.05; simple mode, *p* = 0.454 > 0.05; weighted mode, *p* = 0.401 > 0.05). At the same time, we did not find a significant causal relationship between HF and AD with four of the methods (MR-Egger, *p* = 0.195 > 0.05; IVW, *p* = 0.0879 > 0.05; simple mode, *p* = 0.170 > 0.05; weighted mode, *p* = 0.110 > 0.05), but the WM method indicated a significant effect of HF on AD (*p* = 0.025 < 0.05). Because the statistical powers of IVW and MR-Egger are more than that of WM, we think that there is no causal effect of HF on AD. Sensitivity analysis and horizontal pleiotropy were not detected in the MR analysis.

**Conclusion:** Our results did not provide significant evidence indicating any causal relationships between HF and AD in the European population. Therefore, more large-scale datasets or datasets related to similar factors are expected for further MR analysis.

## Introduction

The occurrence of heart failure (HF) and Alzheimer’s disease (AD) may result in a series of severe medical and financial problems for ordinary families and societies in general. The combination of HF and AD raises the cost of health care, and the present study provides sufficient evidence to show that this increase in cost is approximately fourfold if someone suffers from the combination of HF and AD (Chhatre et al., 2009). In addition, we have found increases in rehospitalization rates and mortality in patients who have been affected simultaneously by HF and memory dysfunction (Vogels et al., 2007; Pressler et al., 2010). In 1997, “cardiogenic dementia” was first proposed by researchers (Lancet, 1977). Since then, a growing body of studies have shown that there exists a close relationship between HF and cognitive impairment (Cohen and Mather, 2007). Along with advances in research techniques and the unremitting efforts of clinicians, there is much persuasive evidence for a relationship between HF and AD due to similar risk and genetic factors, including gender, age, and obesity (Kivipelto et al., 2005; Vina and Lloret, 2010; Roger, 2013). Moreover, genetic evidence also suggests that the Apo E4 (apolipoprotein E4) allele and variants in the presenilin 1 (PSEN1) and presenilin 2 (PSEN2) genes have close contact in the development of HF and AD (Pang and Baum, 2000; Van Uden et al., 2000; Li et al., 2006; Gianni et al., 2010; Iadecola, 2013). In recent years, there has been a growing belief that HF is one of the risk factors for AD (Qiu et al., 2006). Although researchers have found similar mechanistic pathways for HF and AD, there is a lack of sufficient evidence to estimate the causal interrelationships between HF and AD.

Moreover, the causal relationship between HF and AD based on summary-level and genome-wide association study (GWAS) datasets has not been explored. Therefore, exploring this relationship between HF and AD and the causal direction of its effects is essential for clinicians to pursue. By determining these causal relationships, clinicians can provide a relatively accurate prognosis for patients.

The strength of observational studies is that they are rapid and inexpensive to conduct. Moreover, they play an essential role in exploring new hypotheses for the causal relationships existing between exposure and outcomes, which provides important hypotheses for further studies. Observational studies allow for studies of rare diseases in small sample sizes (Lewallen and Courtright, 1998). However, it is well known that observational studies still have several limitations as regards evaluating causal effects that demand prompt solutions and bias in controlling for confounders (Greenland and Morgenstern, 2001). Moreover, because of the inherent weaknesses of traditional observational studies, the estimated effects of HF and AD are subject to certain deficiencies. Underlying these limitations, compared to observational studies, the use of Mendelian randomization (MR), which employs single-nucleotide polymorphisms (SNPs) as instrumental variables (IVs), is essential in order to overcome the limitations of confounders, reverse causality, and bias in observational studies (Davey Smith and Hemani, 2014; Davies et al., 2018).

MR is a persuasive genetic assessment tool that gives a robust estimate of exposures and outcomes (Davey Smith and Hemani, 2014; Davies et al., 2018) when there is a lack of high-quality randomized controlled trials. In MR analysis, instrumental variables evaluate the causal relationships between exposures and outcomes, which need to meet three critical assumptions: 1) genetic variants are strongly associated with exposure, 2) genetic variants are independent of the confounders of exposure and outcome, and 3) genetic variants are related to outcome only via exposure (Davey Smith and Hemani, 2014; Davies et al., 2018). The MR method uses summary GWAS datasets to estimate the causal relationships between exposure and outcome rather than genetic variants from individuals (Hemani et al., 2018). A bidirectional MR determines the causal direction of the relationship between two sets of independent genetic variants related to two phenotypes (Richmond and Davey Smith, 2019). Therefore, we applied a two-sample bidirectional MR method to analyze the causal relationships between and the causal direction of HF and AD.

## Materials and Methods

A two-sample bidirectional MR analysis is shown in Supplementary Figure S1, where panel (A) shows the causal effect of AD traits on HF and panel (B) shows the causal effect of HF traits on AD.

### Data Source

Two GWAS datasets were used for the MR analysis. When allele frequencies and exposure or outcome distributions vary substantially between different ethnic groups, population stratification will arise. Population stratification generates an association between exposure and outcome at the summary level of the population (Larsson et al., 2019). Therefore, in order to avoid the bias of population stratification, we can select ethnically homogeneous groups to further the MR analysis. In order to avoid population stratification, only genetic variants derived from European ancestries were employed in the analysis. The IEU GWAS database was developed by the UK Medical Research Council (MRC) Integrative Epidemiology Unit (IEU) at the University of Bristol (Hemani et al., 2018). As of September 2017, the IEU GWAS database contained 1,673 publicly available GWAS datasets, which are constantly being updated and expanded. The datasets have been applied to MR analysis by numerous researchers (Hemani et al., 2018). All database datasets are publicly available for download and use, and our approach relied on summary-level GWAS data to obtain MR estimates. We obtained datasets for HF and AD derived from the IEU GWAS database. The GWAS dataset for AD came from the Alzheimer Disease Genetics Consortium (ADGC), the European Alzheimer’s Disease Initiative (EADI), the Cohorts for Heart and Aging Research in Genomic Epidemiology Consortium (CHARGE), and the Genetic and Environmental Risk in AD/Defining Genetic, Polygenic, and Environmental Risk for Alzheimer’s Disease Consortium (GERAD/PERADES) and included 63,926 individuals (21,982 cases vs. 41,944 controls) of European ancestry. We also obtained information for 10,528,610 SNPs (Kunkle et al., 2019) (https://gwas.mrcieu.ac.uk). The GWAS dataset for HF was retrieved from the IEU GWAS database, which contained 977,323 individuals of European descent; we also obtained detailed information for 7,773,021 SNPs (Shah et al., 2020) (https://gwas.mrcieu.ac.uk). In order to avoid deviations arising from population stratification, we used only datasets for people with European ancestry.

### SNP Selection

Based on the above two datasets, we formulated a series of quality control standards to screen SNPs that were used as IVs. First, to meet assumption 1 (IVs are strongly associated with exposure), we selected IVs that achieved a genome-wide significance level (*p* < 5 × 10^–8^) from exposures (AD or HF). Second, linkage disequilibrium (LD) reflects the association between alleles at linked loci, which destroys the randomness of genetic variants (Collins, 2009). In the process of data processing, to meet IV assumptions 2 or 3, LD should be removed. Therefore, some parameters were set to eliminate the genetic variants with possible LD, that is, LD was pruned using the default settings of the clump data function of the TwoSampleMR package (physical distance threshold, 10,000 kb; *r* ^*2*^ < 0.001). Third, the PhenoScanner database (Kamat et al., 2019) contains a large amount of data to link SNPs with diseases and phenotypes, and we used the PhenoScanner tool to exclude any of the selected SNPs that were associated with possible mechanistic pathways of outcomes (HF or AD). To test whether the selected SNPs were associated with other risk factors for HF, we input the selected SNPs into the tool one by one (*p* < 5 × 10^–8^ indicated that this SNP was strongly associated with phenotypes) and set the rest of the parameters to default values. Last, the above-selected SNPs with a minor allele frequency (MAF) of <0.01 were also not considered in order to avoid potential bias from the original datasets owing to low confidence. If a proxy variant could not be found for a target variant from the study of the outcome, the variant should be excluded. At the same time, one SNP should be eliminated for being palindromic with intermediate allele frequencies when harmonizing exposure and outcome using the harmonise_data function of the TwoSampleMR package. F statistics were also calculated in order to evaluate the strength of association between SNPs and exposure, and we set the threshold for the F statistic to be > 10. One of the SNPs with a value of <10 was deemed to be a “weak instrument” and was ruled out of our MR analysis (Burgess et al., 2017). The F statistic was calculated using the formula *F* = *β* ^2^/SE^2^, where *β* represents the effect on the risk of exposure and SE is the standard error (Bowden et al., 2016b).

### MR Analysis

In the MR analysis, the statistical analysis was performed using R software version 4.1.0 along with the R packages Two samples used are MR and MR-PRESSO (Hemani et al., 2018; Verbanck et al., 2018), and the codes used in our study are publicly available.

### Statistical Analysis for MR

The IVW method was implemented as the primary MR method for combining the Wald ratios of the causal effects between exposure and outcome, and it can provide the most accurate estimate for all selected SNPs (Burgess et al., 2013). The IVW method was found to be the most predictable if directional pleiotropy is non-existent. The MR-Egger method provides a causal estimate using the slope of the weighted linear regression against horizontal pleiotropy (Bowden et al., 2016b). The weighted median estimator gives a credible estimate of effect when up to half of the weight is derived from valid IVs (Bowden et al., 2016a). The simple mode and weighted mode can also be applied to MR analysis. The five methods mentioned earlier were used in our MR analysis. In bidirectional MR studies, we reverse the functions of exposure and outcome because exposure and outcome are a two-way street. Therefore, we sought to estimate AD based on the risk of HF. At the same time, we performed a bidirectional MR analysis to determine the causal effect of HF on AD.

### Sensitivity Analysis

The existence of heterogeneity among IVs is an indicator of violations of the MR assumptions (Bowden et al., 2017). The IVW and MR-Egger methods were used to estimate the level of heterogeneity among the IVs. Cochran’s *Q* test and the *I* ^2^ statistic calculated the level of heterogeneity among the causal estimates for the selected SNPs using the IVW and MR-Egger methods: *p* < 0.05 and *I* ^2^ > 50% reflect the existence of significant heterogeneity (Zhang et al., 2015). Furthermore, to perform sensitivity analysis, we created a leave-one-out plot (Burgess and Thompson, 2017) to ensure that the effect was not disproportionately reliant on any particular SNP. If the estimated effect changes very significantly when one of the genetic variants is removed, that genetic variant is an outlier or sensitive, and the MR analysis is repeated. Last, the potentially unbalanced horizontal pleiotropy tests among the genetic variants were performed by means of MR-Egger, MR-PRESSO, and funnel plots. For the MR-Egger intercept, *p* < 0.05 is an indicator of genetic variants with directional pleiotropy (Burgess and Thompson, 2017). MR-PRESSO detects the potential pleiotropy, which is adequately powered among even a tiny subset of loci (Verbanck et al., 2018). In addition, MR-Egger regression generally had lower precision than the MR-PRESSO outliner test (Verbanck et al., 2018). The tests of sensitivity mentioned above are two-sided in our bidirectional MR analysis.

## Results

### Effect of AD Traits on HF

In selecting the IVs from the GWAS dataset for AD, we acquired 21 IVs without LD (*r* ^*2*^ < 0.001) that were within the physical distance threshold (10,000 kb) and achieved genome-wide significance (*p* < 5 × 10^–8^). We used the PhenoScanner tool to rule out five SNPs that were associated with potential pleiotropic effects (rs34665982 and rs12151021 are associated with hemoglobin concentration, rs7412 and rs147711004 with coronary artery disease, and rs9381563 with reticulocytes). Of the SNPs mentioned earlier, we detected two SNPs (rs72654445 and rs139136389) not found in HF within the MAF threshold (>0.01). At the same time, two SNPs (rs11257242 and rs114812713) were removed for being palindromic with intermediate allele frequencies by the TwosampleMR function of the R package. After rigorous calculations, the F statistic for individual SNPs ranged from 31.9 to 466.8, and a weak instrument did not exist. By a series of SNP selection quality control steps, 12 SNPs were selected for further MR analysis (Table 1; Supplementary Figure S2A).

**TABLE 1:** Detailed information of selected SNPs for MR analysis of the causal effect of AD on HF.

NO*.*	SNP	Chr	Pos	Effect_allele	Other_allele	MAF	Beta	SE	*p*	F
1	rs1582763	11	60,021,948	A	G	0.3628	−0.1232	0.0149	1.19E-16	68.367
2	rs73223431	8	27,219,987	T	C	0.3459	0.0936	0.0153	8.34E-10	37.426
3	rs6733839	2	127,892,810	T	C	0.3797	0.1693	0.0154	4.02E-28	120.86
4	rs3851179	11	85,868,640	C	T	0.3708	0.1198	0.0148	5.81E-16	65.522
5	rs679515	1	207,750,568	C	T	0.172	−0.1508	0.0183	1.55E-16	67.905
6	rs150685845	19	45,675,180	G	A	0.0129	0.5561	0.0645	6.62E-18	74.334
7	rs3740688	11	47,380,340	T	G	0.4573	0.0935	0.0144	9.70E-11	42.16
8	rs1081105	19	45,412,955	C	A	0.0308	0.942	0.0436	1.51E-103	466.8
9	rs111278137	19	45,215,081	A	G	0.0139	−0.4735	0.0713	3.20E-11	44.102
10	rs12590654	14	92,938,855	A	G	0.337	−0.0906	0.0157	8.73E-09	33.301
11	rs11767557	7	143,109,139	C	T	0.2177	−0.1028	0.0182	1.56E-08	31.904
12	rs867230	8	27,468,503	A	C	0.4016	0.1333	0.0158	3.49E-17	71.178

AD, Alzheimer’s disease; HF, heart failure; SNP, single-nucleotide polymorphism; Chr, chromosome; MAF, minor allele frequency; β, the effect size of AD; SE, standard error; *p*, *p*-value; F, F statistics evaluate the weak instrument variables.

Five methods (MR-Egger, WM, IVW, simple mode, and weighted mode) and 12 significant IVs were employed to analyze the causal effects of AD on HF, which were found to be consistent among the five MR methods, and this result did not suggest a significant association (MR-Egger, *p* = 0.634 > 0.05; WM, *p* = 0.337 > 0.05; IVW, *p* = 0.471 > 0.05; simple mode, *p* = 0.454 > 0.05; weighted mode, *p* = 0.401 > 0.05) (Table 2; Supplementary Figure S3).

**TABLE 2:** Bidirectional MR results between HF and AD.

Methods	Influence of AD traits on HF			Influence of HF traits on AD		
	OR (95%CI)	Beta	*P*	OR (95%CI)	Beta	*p*
IVW	0.985 (0.945, 1.026)	−0.015	0.471	0.848 (0.702,1.025)	−0.165	0.088
MR-Egger	0.982 (0.913, 1.056)	−0.018	0.633	0.665 (0.380,1.161)	−0.409	0.195
Weighted median	0.977 (0.932, 1.025)	−0.023	0.337	0.759 (0.596,0.967)	−0.275	0.025
Simple mode	0.970 (0.898, 1.047)	−0.03	0.454	0.729 (0.483,1.100)	−0.317	0.21
Weighted mode	0.978 (0.932, 1.028)	−0.022	0.401	0.722 (0.505,1.031)	−0.326	0.182

AD, Alzheimer’s disease; HF, heart failure; *p*, *p*-value; IVW, inverse variance weighted.

The heterogeneity test did not show significant heterogeneity among the 12 IVs with the MR-Egger and IVW methods (MR-Egger: *Q* = 16.857, *I* ^2^ ≈ 35% < 50%; *p* = 0.776 > 0.05, and IVW: *Q* = 16.874, *I* ^2^ ≈ 35% < 50%; *p* = 0.112 > 0.05) (Table 3). We complemented the leave-one-out cross-validation to calculate the level of sensitivity; the effects were not disproportionately driven by a particular SNP (Supplementary Figure S4). The test for MR-Egger regression was not significant (MR-Egger intercept = 0.0006, *p* = 0.921 > 0.05), which indicated that the results were not biased by horizontal pleiotropy. A funnel plot was also created to test for the absence of horizontal pleiotropy (Table 3; Supplementary Figure S5). The MR-PRESSO global test and the MR-PRESSO outliner test did not find any heterogeneity or outliners.

**TABLE 3:** Heterogeneity test and horizontal pleiotropy test.

Exposure	Outcome	Heterogeneity test (IVW)			Heterogeneity test (MR-Egger)			Horizontal pleiotropy test (MR-Egger)		
		*Q*	I^2^	*p*-value	*Q*	I^2^	*p*-value	Intercept	SE	*p*-value
AD	HF	16.87	35%	0.112	16.86	35%	0.077	0.0006	0.0061	0.921
HF	AD	5.019	-0.6	0.755	4.192	-0.9	0.757	0.0167	0.0183	0.393

AD, Alzheimer’s disease; HF, heart failure; IVW, inverse variance weighted; SE, standard error.

### Effect of HF Traits on AD

In the bidirectional MR analysis, we reversed the location of exposure and outcome and repeated the steps in *Effect of AD Traits on HF* above. With the LD threshold (*r* ^*2*^ < 0.001, kb = 10,000), we reached the level of genome-wide significance (*p* < 5 × 10^–8^). A total of 12 HF IVs were employed in this study, which were not associated with potential pleiotropy by the PhenoScanner tool. All the 12 selected IVs were found in HF within the MAF threshold (>0.01). However, three SNPs (rs1556516, rs4746140, and rs4766578) were removed from the study for being palindromic with intermediate allele frequencies. The F statistic for individual SNPs ranged from 30.9 to 83.1 (F statistic >10). Thus, nine SNPs were selected for further analysis (Table 4; Supplementary Figure S2B).

**TABLE 4:** Detailed information of selected SNPs for MR analysis of the causal effect of HF on AD.

NO*.*	SNP	Chr	Pos	Effect_allele	Other_allele	MAF	Beta	SE	*p*	F
1	rs11745324	5	137,012,171	A	G	0.2296	−0.0528	0.0095	2.34E-08	30.89
2	rs660240	1	109,817,838	C	T	0.1988	0.0611	0.0097	3.25E-10	39.677
3	rs56094641	16	53,806,453	G	A	0.4344	0.0454	0.008	1.21E-08	32.206
4	rs1510226	6	160,816,409	C	T	0.0109	0.162	0.0285	1.27E-08	32.31
5	rs600038	9	136,151,806	C	T	0.2147	0.0569	0.0096	3.68E-09	35.13
6	rs17617337	10	121,426,884	T	C	0.2177	−0.0561	0.0095	3.65E-09	34.872
7	rs55730499	6	161,005,610	T	C	0.0755	0.1058	0.0157	1.83E-11	45.412
8	rs4135240	6	36,647,680	C	T	0.3439	−0.0486	0.0084	6.84E-09	33.474
9	rs17042102	4	111,668,626	A	G	0.1501	0.1103	0.0121	5.71E-20	83.096

AD, Alzheimer’s disease; HF, heart failure; SNP, single-nucleotide polymorphism; Chr, chromosome; MAF, minor allele frequency; β, effect size of AD; SE, standard error; *p*, *p*-value; F, F statistics evaluate the weak instrument variables.

There was no evidence suggesting a significant association between HF traits on AD using the four methods (MR-Egger, *p* = 0.195 > 0.05; IVW, *p* = 0.088 > 0.05; simple mode, *p* = 0.170 > 0.05; weighted mode, *p* = 0.110 > 0.05). However, the WM method indicated a significant effect of HF on AD (*p* = 0.025 < 0.05). The IVW and MR-Egger methods were more persuasive than the WM method (Bowden et al., 2016b). Therefore, we believe that HF does not have a causal effect on AD (Table 2; Supplementary Figure S6).

The MR-Egger and IVW methods revealed no heterogeneity among the nine IVs (MR-Egger: *Q* = 4.192, *I* ^2^ ≈ −0.90 < 50%, *p* = 0.757 > 0.05 and IVW: *Q* = 5.019, *I* ^2^ ≈ −0.60 < 50%, *p* = 0.755 > 0.05) (Table 3). We did not find that the effects were driven by any SNPs with the leave-one-out cross-validation (Supplementary Figure S7). As for horizontal pleiotropy, we conducted MR-Egger regression and created a funnel plot (MR-Egger intercept = 0.017, *p* = 0.393 > 0.05), which did not reveal any significant results (Table 3; Supplementary Figure S8). Moreover, we did not find the presence of heterogeneity and outliners when we used the MR-PRESSO global test and the MR-PRESSO outliner test.

## Discussion

To the best of our knowledge, our study is the first to use a two-sample and bidirectional method to determine whether there is a causal relationship between heart failure (HF) and Alzheimer’s disease (AD) in European populations. Although we applied a series of quality control steps in our analysis, the results of our study did not support the hypothesis that there is a meaningful causal relationship between genetically predicted HF and AD because the statistical analysis did not provide sufficiently conclusive results to support the hypothesis. On the one hand, the conclusions of previous observational epidemiological studies suggesting a significant association between HF and AD may have resulted from the presence of uncontrolled biases or cofounders; on the other hand, because of the lack of sufficient GWAS studies of HF and AD, our results need to be carefully explained. In addition, our MR analysis indicates only an average lifetime risk, and it cannot explain whether the existence of HF (or AD) in a particular period has any effect on the level of risk of AD (or HF). Moreover, with regard to the selected IVs for AD or HF used in this study, it is the biological correlation with HF or AD that is important, and the biological actors in this case are still imprecisely known. Moreover, the selected SNPs had limitations and we explored only a small part of the variance in HF and AD, although we used several methods to exclude horizontal pleiotropy and weak instrument bias.

To ascertain the causal relationships between AD and HF, obtaining large-scale GWAS datasets derived from AD or HF may be effective. Moreover, this study included only European populations and not other ethnic groups, so causal relationships between HF and AD may well be found when other ethnicities (African and Asian) are examined. Moreover, in observational studies, there are many similar risks and genetic factors that provide relevant evidence to prove an association between HF and AD, for example, obesity, age, Apo E4, etc. Therefore, in future MR studies, valid results may be obtained if we conduct further MR analysis on large-scale summary-level GWAS datasets of risk factors (age and obesity) or genetic factors derived from Apo E4.

Overall, in this bidirectional MR study, we failed to find persuasive evidence suggesting a causal relationship between genetic liability to AD and HF. In future studies, in order to explore the relationship between HF and AD, high-quality clinical trials and excellent laboratory studies will be indispensable. From the researcher’s point of view, MR is a relatively superior method for understanding the relationships between various diseases (Wootton et al., 2018). We hope that this method will improve the quality of the control steps.

## Data Availability

The datasets presented in this study can be found in online repositories. The names of the repository/repositories and accession number(s) can be found in the article/Supplementary Material.
