# Supplementary material for: Causal Association Between Heart Failure and Alzheimer’s Disease: A Two-Sample Bidirectional Mendelian Randomization Study
Source: Front Genet. 2022 Jan 11;12:772343. doi: 10.3389/fgene.2021.772343 (PMC8787319; doi:10.3389/fgene.2021.772343)
Supplement: Supplementary file 1 [file Presentation1.PPTX]

## Slide 1
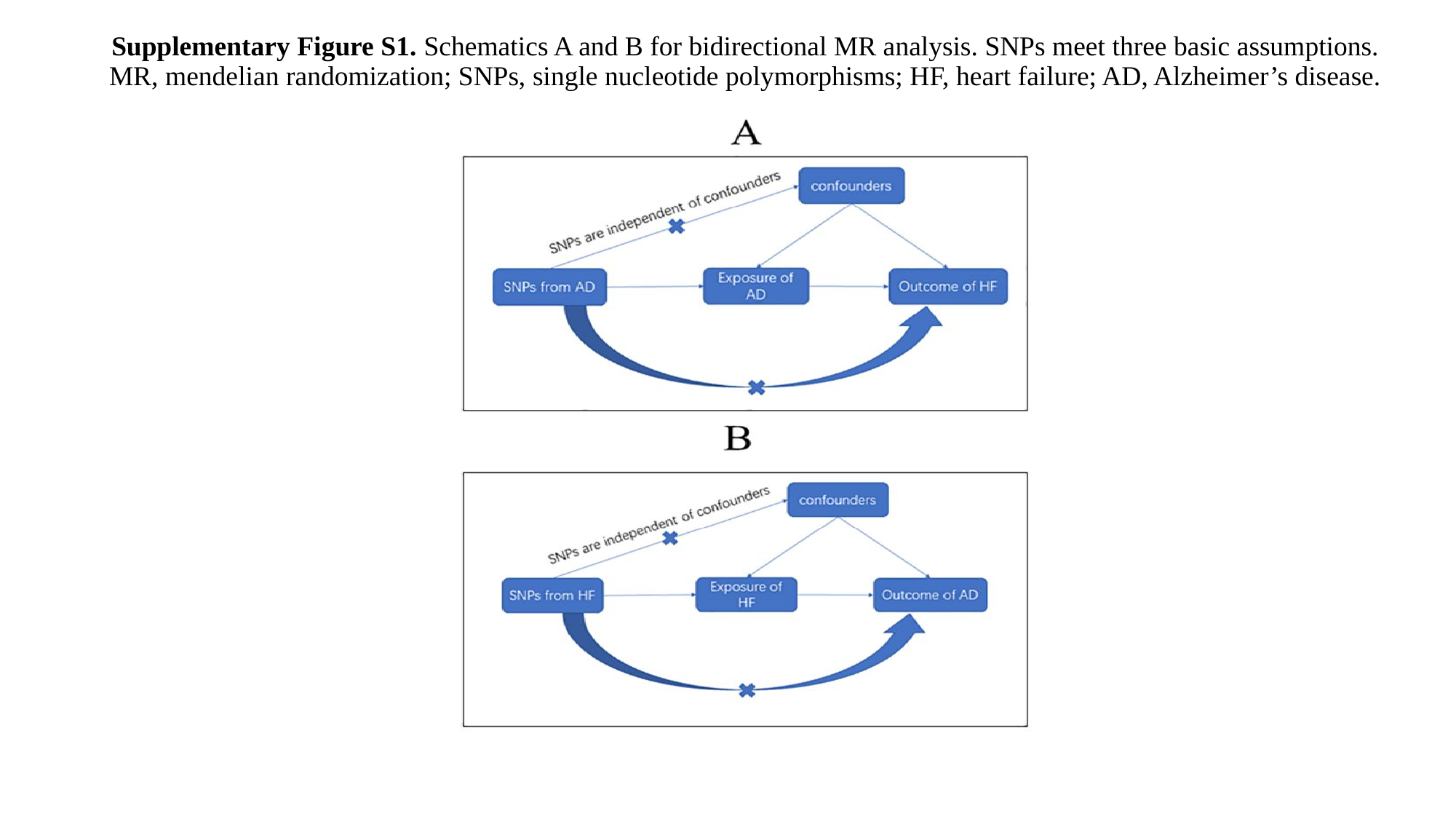

Supplementary Figure S1. Schematics A and B for bidirectional MR analysis. SNPs meet three basic assumptions. MR, mendelian randomization; SNPs, single nucleotide polymorphisms; HF, heart failure; AD, Alzheimer’s disease.

## Slide 2
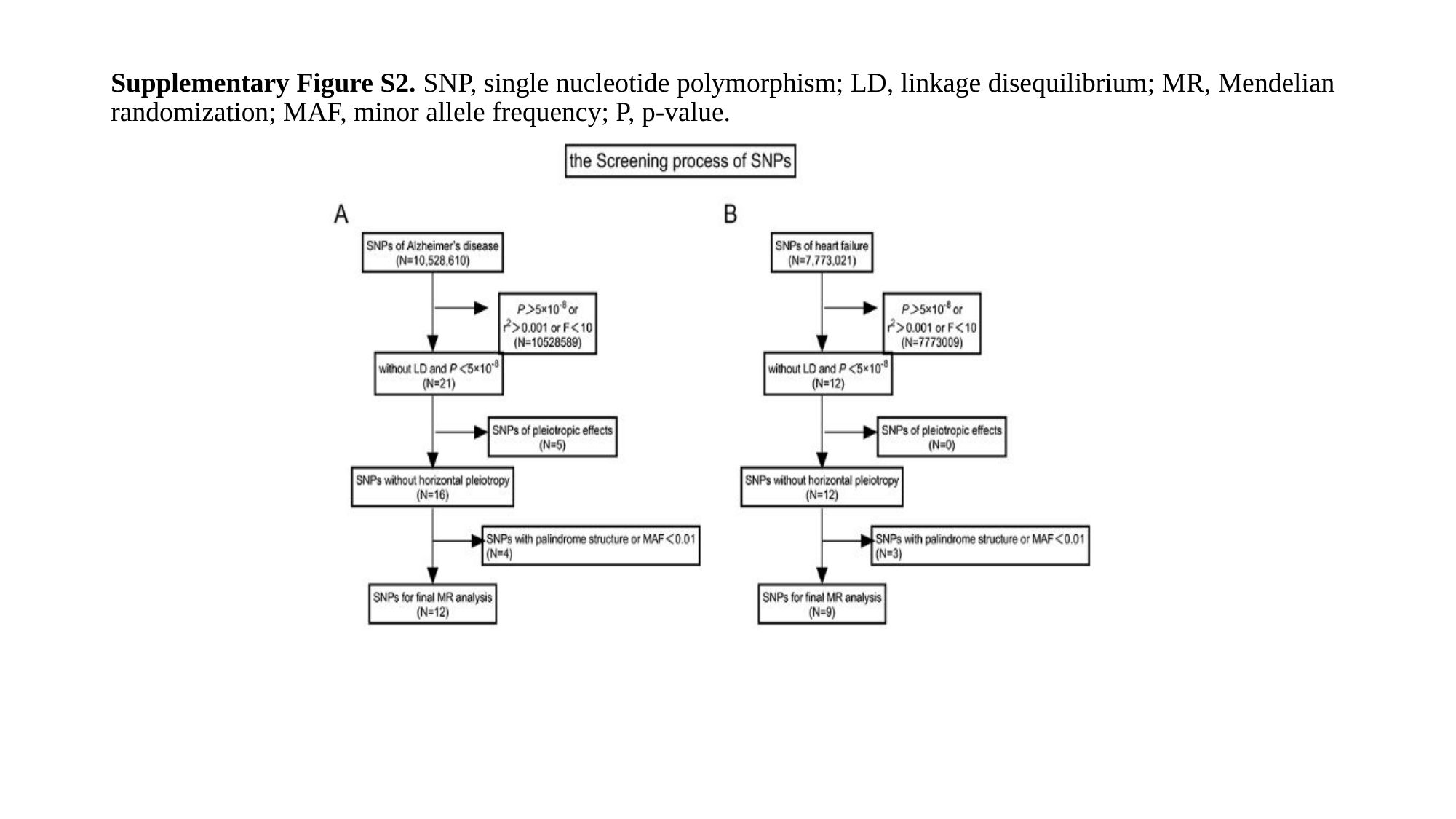

# Supplementary Figure S2. SNP, single nucleotide polymorphism; LD, linkage disequilibrium; MR, Mendelian randomization; MAF, minor allele frequency; P, p-value.

## Slide 3
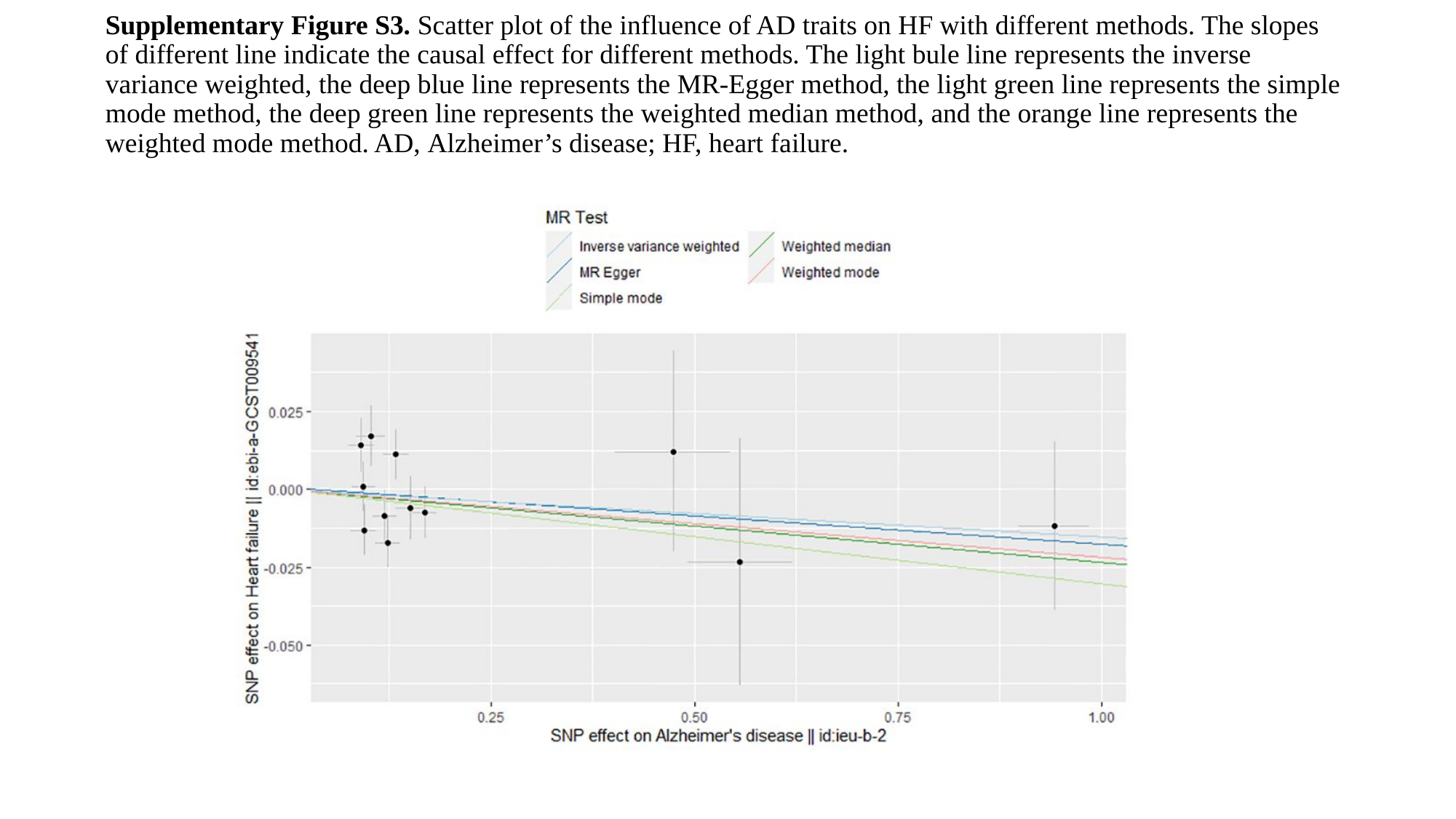

# Supplementary Figure S3. Scatter plot of the influence of AD traits on HF with different methods. The slopes of different line indicate the causal effect for different methods. The light bule line represents the inverse variance weighted, the deep blue line represents the MR-Egger method, the light green line represents the simple mode method, the deep green line represents the weighted median method, and the orange line represents the weighted mode method. AD, Alzheimer’s disease; HF, heart failure.

## Slide 4
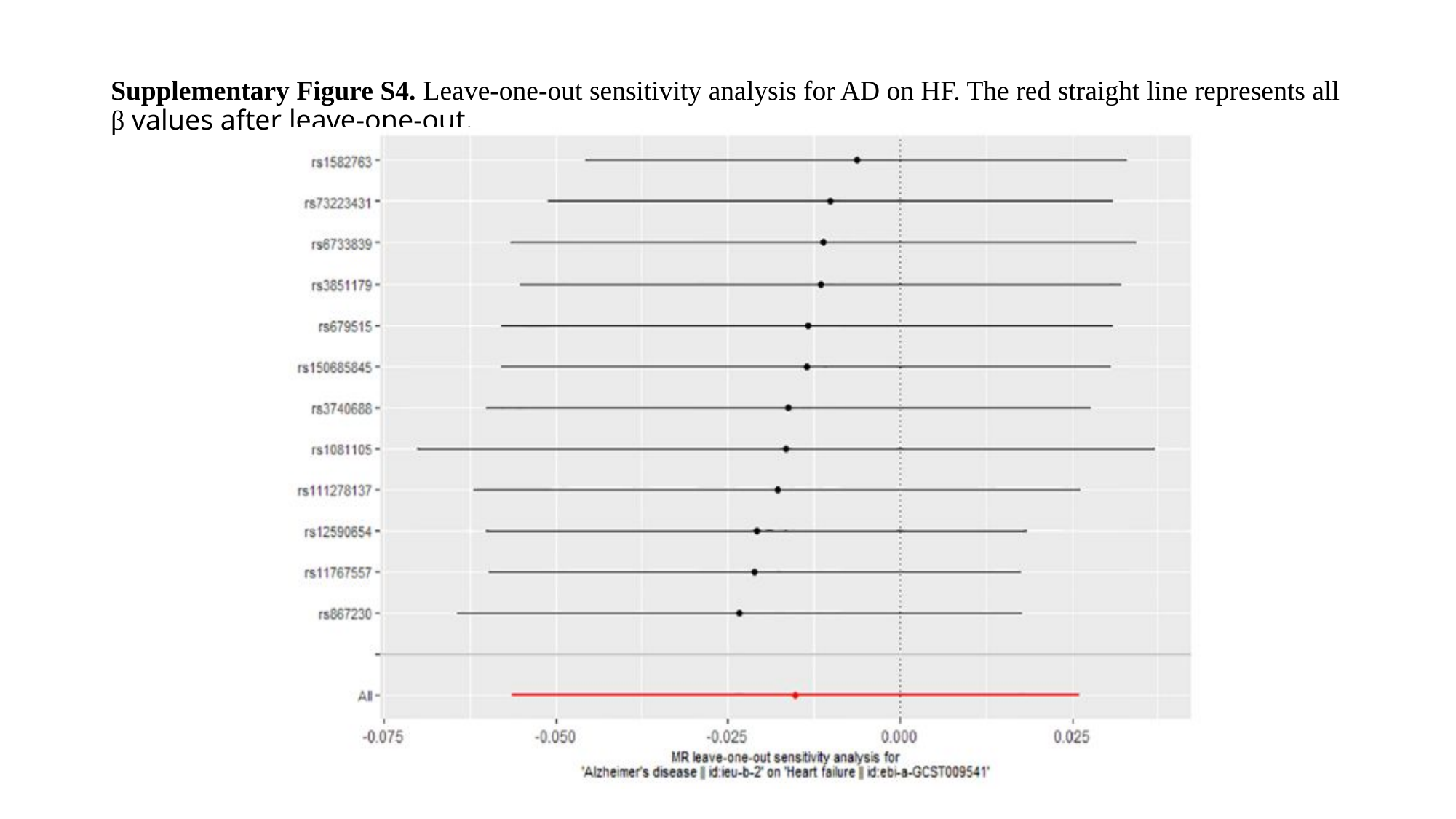

# Supplementary Figure S4. Leave-one-out sensitivity analysis for AD on HF. The red straight line represents all β values after leave-one-out.

## Slide 5
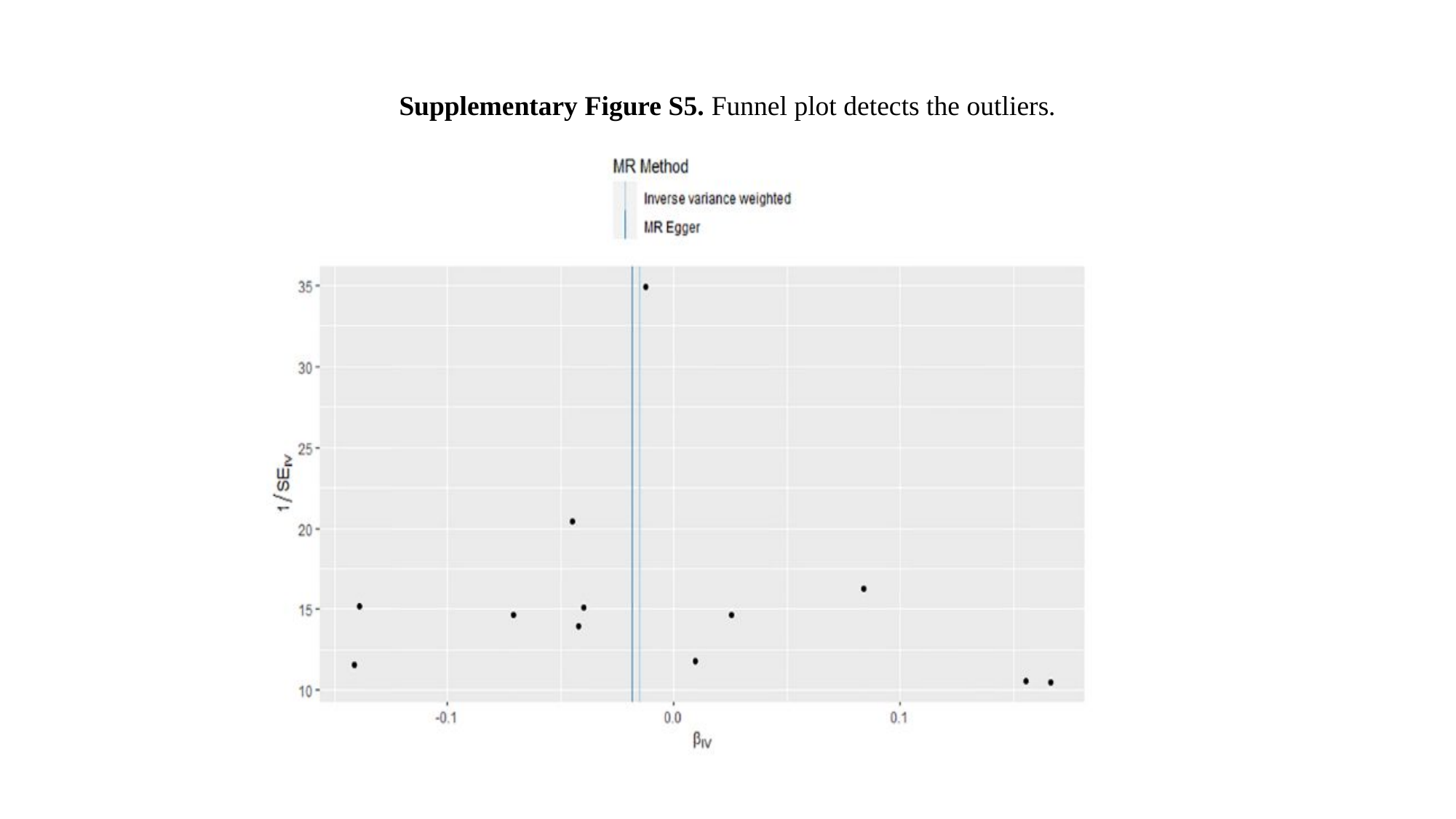

# Supplementary Figure S5. Funnel plot detects the outliers.

## Slide 6
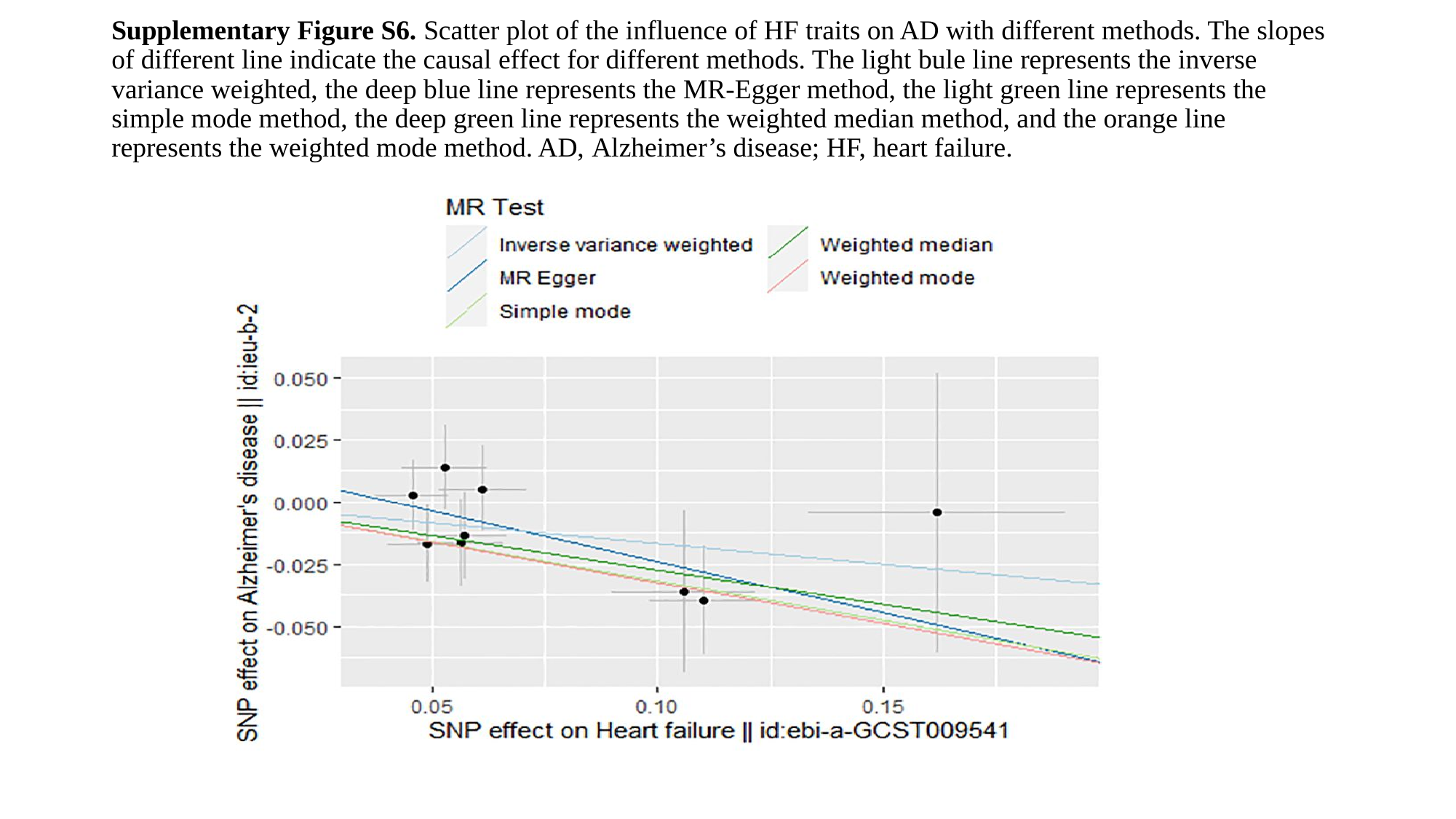

# Supplementary Figure S6. Scatter plot of the influence of HF traits on AD with different methods. The slopes of different line indicate the causal effect for different methods. The light bule line represents the inverse variance weighted, the deep blue line represents the MR-Egger method, the light green line represents the simple mode method, the deep green line represents the weighted median method, and the orange line represents the weighted mode method. AD, Alzheimer’s disease; HF, heart failure.

## Slide 7
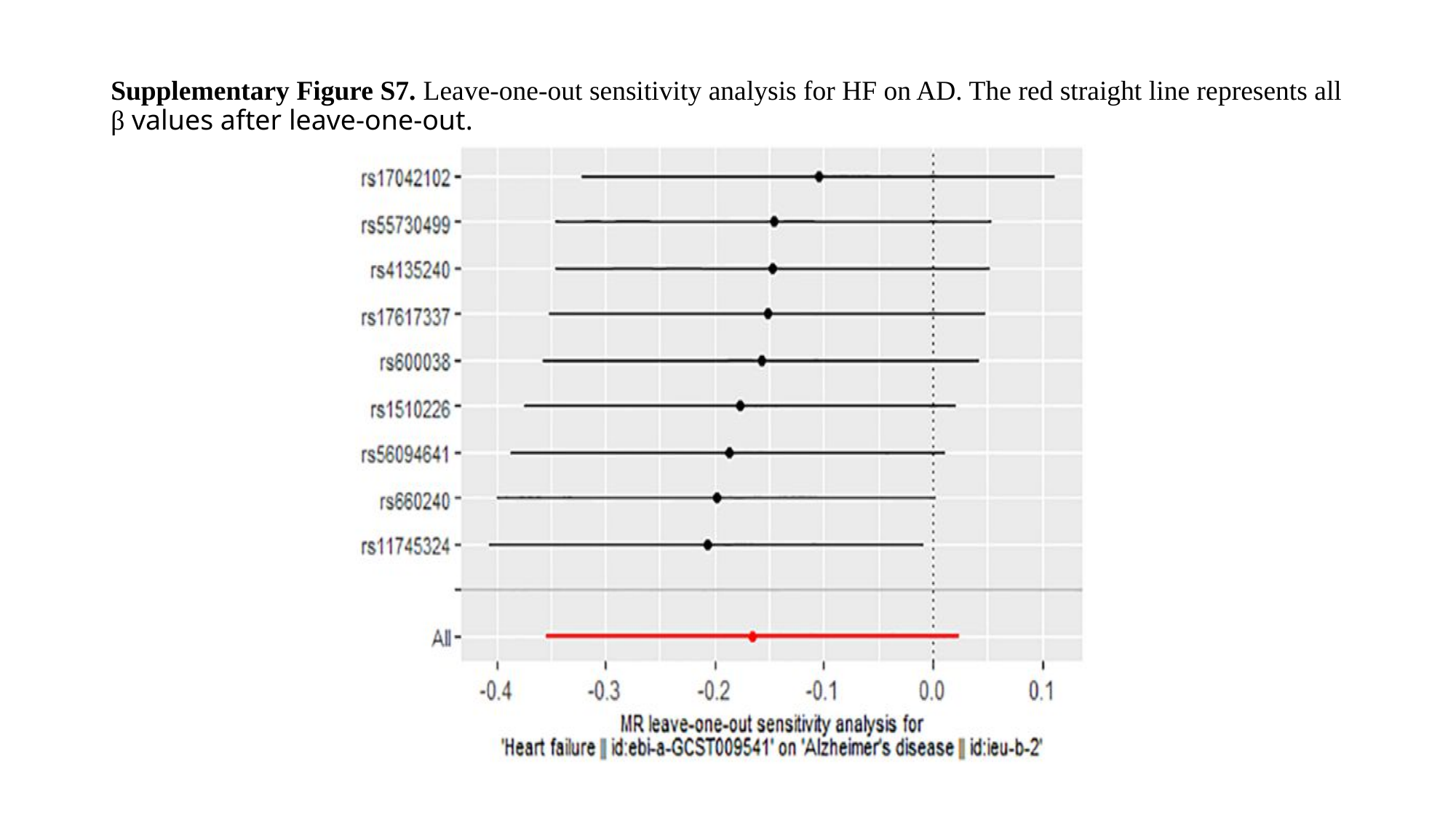

# Supplementary Figure S7. Leave-one-out sensitivity analysis for HF on AD. The red straight line represents all β values after leave-one-out.

## Slide 8
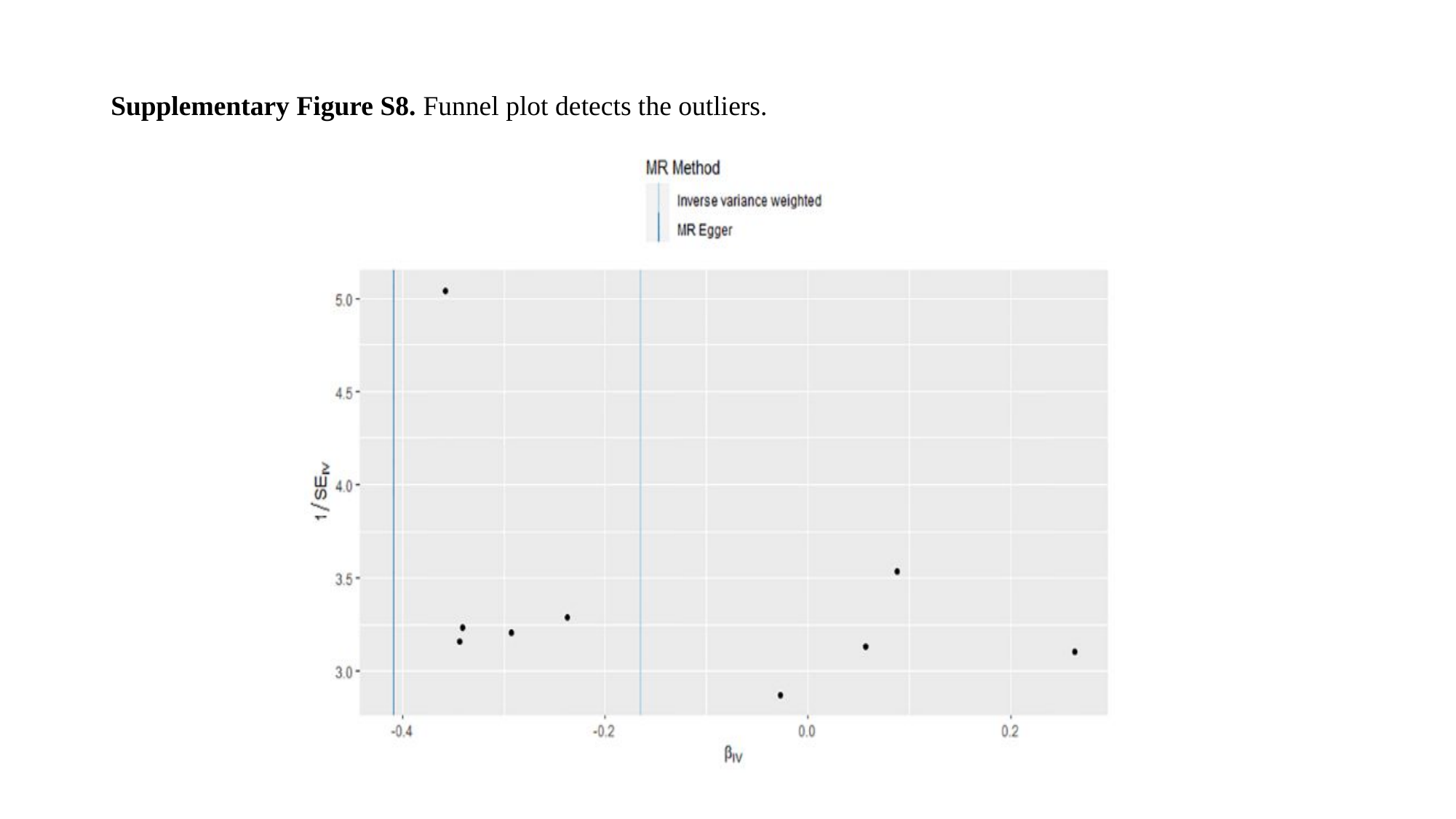

# Supplementary Figure S8. Funnel plot detects the outliers.
